# “Do I need to reinvent myself?” Stigmatization of sport-related identities of Chinese students

**DOI:** 10.3389/fpsyg.2024.1386796

**Published:** 2024-12-09

**Authors:** Jiangxi Chen, Weiguang Ni

**Affiliations:** The Physical Education College of Jilin University, Changchun, China

**Keywords:** student-athletes, stigma, sports students, stereotype threat, identity

## Abstract

**Objectives:**

While many studies have shown that student-athletes are a stigmatized group and topics related to them have received increasing attention, few studies have focused on the current situation of Chinese sports students (*tiyu sheng*) with the same dual identity as student-athletes. Thus, this study aims to understand and recognize the negative perceptions that students with sports identities experience in the Chinese educational and cultural context and the impact of these perceptions on them.

**Methods:**

This qualitative study used an interpretive description methodology to collect data through semi-structured, in-depth interviews conducted by the researcher with 11 sports students, 4 regular students, 3 teachers, and 2 parents. Then, the interview data was analyzed using reflective thematic analysis.

**Results:**

Three themes were eventually extracted: (1) The onset and development of stigma, (2) Stigma factors in the spotlight, and (3) the Response to stigmatization by sports students.

**Conclusion:**

Findings indicated that sports students are subjected to various stigmas under the negative filters of society. The stigmas suffered by sports students vary across school years and levels, and their perceptions of stigmas may also change over time and in different environments. In addition, being subjected to stereotype threat and self-stigma may have hurt some participants. The study’s results also provide evidence that the Internet plays a vital role in exacerbating the development of stigmatization among sports students, which needs to be determined by further research. These findings will contribute to subsequent research on the de-stigmatization of sports students.

## 1 Introduction

The term stigma first referred to the Greeks’ use of body marks to signify something morally abnormal or wrong, which were painted or carved on the human body to indicate that they were slaves, criminals, traitors, etc. Later, the term was extended to include all marks or symbols that perceive or infer deviations from the normative situation. Sociologist E. Goffman attributed “stigma” to people who deviate from social norms and used stigma as a starting point for social discrimination (Bos et al., 2013). According to him, stigma is a derogatory, insulting label that society applies to individuals or groups of people because they have some socially undesirable or disreputable characteristic that lowers their status in society. Stigma is formally divided into abominations of the body, such as physical defects, physical disabilities, etc.; blemishes of individual characters, such as mental illness, alcoholism, etc.; and tribal identities, such as race, gender, religion, etc. (Goffman, 1986).

Furthermore, stigma is a state of entitlement in which elements of labeling, stereotyping, isolation, and status deficit coexist, and there is an overlay of various stigmatizing elements in this state (Link and Phelan, 2001). Consequently, stigmatized people also predominantly suffer from other harmful behaviors of others, such as stereotyping. Stigmatization, on the other hand, is a dynamic process by which one group imposes and maintains human inferiority on another group, characterizing the group’s qualities that are biased toward the negative and thereby overshadowing other characteristics, becoming an indicator that corresponds to the group’s attributes in an essential sense (Goffman, 1986). This process is one of the most common strategies employed by the party in a position of strength and without stigma (Kurzban and Leary, 2001).

Stigma occurs in many areas of society; for example, some people tend to believe that black athletes are less intelligent than white athletes because of their genetic predisposition to athletic performance, and even that black people are often encouraged to participate in athletic training and athletic competition more often than in intellectual endeavors (Moskowitz and Carter, 2018). Globally, many individuals who participate in both competitive sports and higher education have dual career identities in sport and education, often referred to as “student-athletes” in the scientific literature (Steele et al., 2020). This group seems to have a prejudice-prone “paradox” in its intersecting identities, as the general public perceives education and academics as generally related to intelligence (De Castella and Byrne, 2015), while some still maintain the stereotype of the “Dumb Athlete” (Wininger and White, 2015). Even the historical origins of this bias can be traced back to 500 B.C. when Greek athletes were blamed for spending too much energy on competition while neglecting their intellectual development and were described by some philosophers of the time as useless, ignorant, and mentally retarded citizens (Sailes, 1993). In fact, student-athletes have also been subjected to various stigmas (Simons et al., 2007). and they are usually perceived as less intelligent, less motivated, less academically inclined, less willing to prepare for the classroom, and even alcoholic and violent than the average student (English and Kruger, 2016; Levine et al., 2014; Hsu and Li, 2022; Nekvasil, 2018).

The stigma or stereotypes experienced by student-athletes are more related to the “conflictual” nature of their dual identities (Steele et al., 2020; Lu et al., 2018). Identity is one of the aspects of an individual’s broader self-concept that encompasses an individual’s subjective assessment of who they are and how they fit into relationships with others and the social world (Brewer and Gardner, 1996; Caza et al., 2018). Identity salience refers to the subjective importance of identity relative to other identities, and highly salient identities are more likely to be activated in different contexts and motivate behaviors consistent with that identity (Stets and Burke, 2000). Salient self-identities are often developed around key life roles that reflect levels of commitment and investment in those roles (Stryker and Burke, 2000). Athlete identity is the degree to which an individual identifies with an athlete’s role. Athlete identity is a cognitive structure and a social role (Brewer et al., 1993). When athletes are placed in a sports system, they may not engage in exploratory behaviors or have a lower need to participate because they value sports. Their needs for wellbeing and competence are met through sports participation, such as the recognition they can receive from their peers for sports participation and the intrinsic and extrinsic rewards that come with athletic achievement (Brewer and Petitpas, 2017). Academic identity has been defined as the degree to which an individual identifies with the role of a student and is associated with positive outcomes such as goal commitment, commitment to the institution, and persistence in academics (Bowman and Felix, 2017). Regarding student-athletes, studies have found a negative correlation between athlete identity and student identity (Sturm et al., 2011; Yopyk and Prentice, 2005). The contrasting nature of these two identities allows student-athletes to receive special attention.

Stigmatized individuals possess (or are believed to have) specific attributes and traits, and the social identity conveyed by these attributes is devalued in particular contexts (Crocker and Major, 1989). These social identities represent different social groups, and in group categorization, members of the same group are perceived to be more similar than the actual criteria of division. In contrast, different groups are perceived to be more different, which leads to the creation of in-groups and out-groups (Tajfel, 1969). The distinction between in-groups and out-groups may lead to perceptual biases in the “us” and “them” dynamics (Cunningham et al., 2008). In addition, social identity theory explains these stereotypes and stigmas (Hawley et al., 2014), which suggests that people develop their social identities through three main cognitive processes: categorization, social identification, and social comparison. These three stages lead people to categorize themselves and others into a social category or group, give emotional meaning and value to the identification with a particular group, and finally, compare the group they belong to with other groups (Al Ramiah et al., 2011). For such comparisons, people are always more inclined to maintain the superiority of their in-group (Scheepers and Ellemers, 2019). Student-athletes have more athlete identities than ordinary students, and they are naturally divided by the outside world into a separate group distinct from the student body. The perceived bias (prejudice and negative stereotyping of athletes) caused by the group division (Geary et al., 2024), coupled with society’s reinforcement of the non-athlete group’s identity, may further create a stigma against student-athletes.

The stigma of student-athletes caused by various labels and negative stereotypes can have a negative impact on them. For example, studies have found that discrimination and stereotypes are stressors that negatively impact the mental health of student-athletes (Tran, 2021). They may feel stressed by balancing the roles of student and athlete because they are often categorized as unintelligent in their learning environment (Lu et al., 2018). These pressures and negative influences contribute largely to their poor academic performance (Stone et al., 2012; Dee, 2014), and this outcome may further exacerbate the vicious cycle of stigma. Additionally, many student-athletes report that their sense of belonging on campus is almost exclusively related to their experiences as athletes and on sports teams (Slaten et al., 2020). Athletic identity faces challenges that impact wellbeing, exacerbated by narrow identity development and stereotypes (Geary et al., 2024). Many scholars have been working to understand the situation of student-athletes and make targeted proposals to reduce these negative impacts (English and Kruger, 2020; Leimer et al., 2014).

However, few studies have focused on the Chinese student population, which resembles student-athletes. This group possesses the same dual identity as the student-athletes defined in the previous studies, except that in addition to their student identity, they also have a sports identity that includes athletes. In China, they are called sports students (*tiyu sheng*). From the designation, their sports identity is more in the spotlight, and thus, they are likely to be classified as a student group with distinctions like the student-athletes in the other studies. At the same time, the associated stigma, stereotypes, etc., would also happen to them. Coupled with the fact that China’s society, culture, and education system have their particularities, the encounters of the group of sports students in this context may be even more complex. Therefore, this study examines how students with sport-related identities in China are perceived and treated by the society and people around them and their situation in this context. Understanding and analyzing their experiences in this regard will provide a basis for strategies to reduce the threat of negative stereotypes and de-stigmatization and promote action for educational reform.

## 2 China’s system of admission to universities through sport and students’ sport-related identities

In China, there are four main ways to be admitted to college through athletic specialties (The following statements refer to the relevant current documents of the Ministry of Education of China and the General Administration of Sport of China, as well as the actual enrollment patterns of sports students): (1) The unified examination for physical education and sports. Such candidates need to have sports specialties, but they have no athlete-level restrictions. In addition to participating in the Gaokao, they must also participate in the sports professional examination in their provinces. (2) The sports single-entry examination. This is an examination organized by the State for the admission of athletes to specific sports majors in various colleges, and students taking this type of examination must reach the technical level of national Division II athletes (inclusive) or above. This kind of examination includes a separate academic examination and a sports test. (3) Enrolment of high-level sports teams in university (high-level athletes). Candidates taking this type of examination can pursue other non-sports majors, such as law, but they must have obtained the technical level of national Division I athletes (or above). (4) Excellent athletes are guaranteed admission to universities (guaranteed admission). This challenging way requires athletes to reach the technical level of top-notch national/international players, break records, or achieve the corresponding rankings in specific large-scale events (e.g., the Olympic Games) to be eligible for exemption from admission.

Therefore, in China, the student-athlete title is only generalized to some students with athletic identities. General students who take the unified examination for physical education and sports do not obtain an athlete-level qualification in high school, and they need to acquire academic knowledge just like regular students. When they enter the university, they mainly have the theoretical knowledge of sports and the fundamental sports skills required. Most of them do not need to maintain regular training and participate in frequent competitions, so compared with the athlete’s identity, they show more of a student’s identity, only that their life and study are more related to sports. Not every student who engages in sports can be a student-athlete, the different levels represent different sports experiences, but in China, they all share a common sports identity—sports students (*tiyu sheng*). Clarifying the relationship between athlete, student, and sports identities will help us better understand the stigmatized situation of sports students.

## 3 Study design

### 3.1 Materials and methods

Interpretive description (ID) was employed as the methodological approach (Thorne, 2016). The goal of ID is not to focus on culture as in ethnography, lived experience as in phenomenology, or theory construction as in grounded theory (Thompson Burdine et al., 2021). The approach aims to provide a detailed description of experience in accessible language to discover and understand the perspectives and worldviews of a phenomenon, a process, or the people involved (Caelli et al., 2003). It has its own set of philosophical underpinnings to help the researcher go about how to study the phenomenon (Bradshaw et al., 2017). Meanwhile, ID belongs not only to the pragmatist paradigm, consistent with a broad epistemological perspective, but also to the interpretivist paradigm, which is based on relativist ontology and constructivist epistemology (Thorne, 2016). In other words, it is firmly focused on answering practical research questions that arise from real-world problems and, through the interactions between participants and researchers, allows for an understanding of the subjective and multiple realities that people construct and the shared experiences that may exist in them (Hamer et al., 2023). Furthermore, any claim made using ID is a tentative truth that can be revised and modified in the future and does not represent the only definitive truth (Uzzell et al., 2022). ID, as a well-established methodology, can gather detailed descriptions of the phenomenon for this study’s exploration of the stigmatization of sports students and help us to get closer to the center of the event through in-depth analyses and explanations of the cause and effect of the phenomenon’s emergence.

Reflective Thematic Analysis (RTA) was used to analyze the interview data in this study because this method is suitable for analyzing data from multiple data sources and seeks to generate patterns of shared meaning around specific themes or ‘central organizing concepts’ (Braun and Clarke, 2013). Thus, RTA as an analytical method fits well with the aim of ID research to explore shared meanings in individual experiences (Thorne, 2016; Hamer et al., 2023; Uzzell et al., 2022).

### 3.2 Participants

Firstly, to ensure that the primary research subjects have suffered from stigma, we extracted the main negative views about stereotypes and stigma from topics about discussing sports students in online forums and then created a questionnaire accordingly to investigate whether sports students have suffered from these negative behaviors (it will not be specific to privacy, only yes or no answers are required), and then finally recruited subjects willing to participate in the study from among those who were willing to fill in the questionnaire and had actually experienced these encounters. Consideration will be given to inviting sports students from different stages of study and levels to participate to ensure heterogeneity within the sample.

In addition to students with a sports student identity, it was decided to collect data from fellow students, teachers, and parents, as it was felt that they would be able to provide further insights into the situation of sports students, particularly around perceptions of the stigma attached to the sports-students identity. Therefore, all of the following were considered for inclusion in the study: (1) students who studied in the same classes as the sports students but did not engage in sports, (2) teachers who taught them theory, and (3) parents of students who were currently engaged in sports in high school or college. Finally, formal interviews were conducted with 11 sports students, 4 average classmates, 3 teachers, and 2 parents (Table 1 for details).

**Table 1:** The demographics of different participants.

Sports-students group				Other group			
Work	Number	Gender	Level	Type	Number	Gender	Work
HS (Age: 16–18)	1	M	I	Mates	1	M	HS
	2	F	II		2	F	HS
	3	M	NO		3	M	UG
	4	M	NO		4	F	UG
UG (Age: 19–22)	1	F	I	Teachers	1	M	HS
	2	F	I		2	F	HS
	3	M	II		3	M	UG
	4	M	NO	Parents	1	M	HS (child)
	5	M	NO				
PG (Age: 23–25)	1	M	NO		2	F	HS (child)
	2	M	NO				

HS, high school; UG, undergraduate; PG, postgraduate; M, male; F, female; I, division I athlete; II, division II athlete; NO, not obtained.

### 3.3 Data collection

Written consent was obtained from all participants before the interviews. Semi-structured interviews were conducted with 20 participants either face-to-face in a private space or by telephone (The interview guide is detailed in Tables 2, 3). The primary interviews were guided by existing literature that served as a theoretical scaffold for the ID research proposal (Yukhymenko-Lescroart and Sharma, 2022; Simons et al., 2007). At the same time, we used a responsive interview style, which means that participants were allowed to take some control over the direction of the conversation, thereby exploring new areas that were not included in the interview guide (Rubin and Rubin, 2011). Questions were asked using as much neutral language as possible to minimize potential bias and preconceptions. The entire interview was transcribed into text and ranged in length from 28 to 67 min (M = 45.6 min; SD = 12.39).

**Table 2:** Schedule used to guide interviews (for other group).

Topic areas	Questions
Perception of sports students	• What is your image of sports students?
	• Do you have much contact with sports students? What do you think is the difference between being around sports students compared to regular students?
	• Do you think it’s easier for sports-students to be admitted to college?
	• Why do you support your children in sports? Is it because they are athletically gifted or something else? (for parents only)
	• How do sports students perform academically differently from the typical students? Do you ever pay special attention to them? (for teachers only)
	• Do you have a sports student friend or would you choose them as your friend? What strengths and weaknesses do you care about them? (for classmates only)
Perception of stigma	• Are you aware of the various stigmas regarding sports students? Is there anyone around you who resents sports students?
	• Where do these stigmas often occur? What are the main areas where these bad perceptions are centered?
	• Do you think the sports students around you fit these bad images? What are your thoughts on these stigmas?
	• What do you think should be done to de-stigmatize the sports student community?

**Table 3:** Schedule used to guide interviews (for sports students).

Topic areas	Questions
Basic information	• How long ago did you start engaging in sports? Reasons for choosing to pursue sports?
	• Through which channel did you enter university? (for undergraduate students only)
Perception of identity	• How do you perceive the sports students? What does sports student status mean to you?
	• Do you find yourself different from the regular students in any way?
	• What words would you use to describe your personality, emotions, behavior, interpersonal relationships and learning?
	• Can you describe what you think the average student looks like?
Daily situation	• How is your daily routine organized?
	• Do you have a lot of non-sporting friends?
	• What is your image in your family? Do they expect more from you?
	• What kind of difficulties do you often encounter? Who do you turn to for help?
Experience of stigma	• How do you think the average students perceive sports students? What misconceptions or bad treatment have you suffered from them?
	• What was your teacher’s attitude toward you? Have you ever received a bad opinion or benefit from a teacher? (e.g., grades)
	• Besides school, have you suffered from being viewed or treated badly in society? Where do these experiences often occur?
Cognition of identity stigma	• Are there people around you who fit these “labels”? (sports students or not)
	• How do you perceive and deal with these prejudices? How has the stigma created by these labels and negative stereotypes affected your studies and life?
	• What do you consider to be your shortcomings? Any attempts to change and make up for it?
	• What are some things you think you would want people around you to know about you? And why?
	• What are your future plans?

In addition to formal interviews, data were collected through observation and informal conversations, and this methodological triangulation helped overcome the limitations of a single data collection method (Thorne, 2016). Observations and informal interviews are conducted with sports students, classmates, and teachers. Most of this part of the process takes place in high school or college classes and the daily lives of the sports students and will include some club activities and job fairs.

### 3.4 Data analysis

The analyzing process was carried out using the following six main stages (Braun and Clarke, 2019). The first stage is familiarizing data, which involves reading the transcripts repeatedly and focusing on the respondents’ emotions and the research topic to get an overall sense of the situation. The second stage is generating initial codes; we need to read the transcript line by line, emphasizing the parts of the content relevant to the research question and assigning descriptive codes to them. The third stage was generating themes/theme development, which involved grouping codes under different potential themes. The fourth stage is reviewing potential themes, which we must consider: (1) Does the theme have a “central organizing concept”? (2) Is there enough meaningful data to support the theme? (3) Is the theme related to our research question? The fifth stage is refining, defining, and naming the theme; at this point, it is necessary to ensure a manageable amount of overlap between theme and theme. At the same time, its title should adequately reflect the sub-themes in the theme. The sixth stage combines appropriate data extraction to present the theme coherently and consistently with an analytical narrative that addresses the current research objectives. Additionally, observational and informal interview data were added to the analysis process to contextualize the themes and whether they were meaningful.

### 3.5 Ethics

Several ethical issues were seriously considered throughout the study. First, the researcher prioritizes building relationships with program participants. This was done to get to know the participants and build trust so that they would understand that we sincerely wanted to reach out to the truth and speak out on behalf of those undesirable phenomena so that they would feel comfortable participating in the interviews. Second, after each interview, all recordings of the conversations and their identifying information are kept strictly confidential; the tapes are transcribed, anonymized, and assigned a separate research pen name to protect each participant’s identity. Also, the interviews will avoid prejudicial remarks and endeavor to make the participants feel safe and comfortable during the conversation. Moreover, the researcher will indicate the purpose in advance when conducting observations or informal conversations, and the person who expresses their views has the right to decide whether to include them in the research.

## 4 Findings

**Figure 1:**
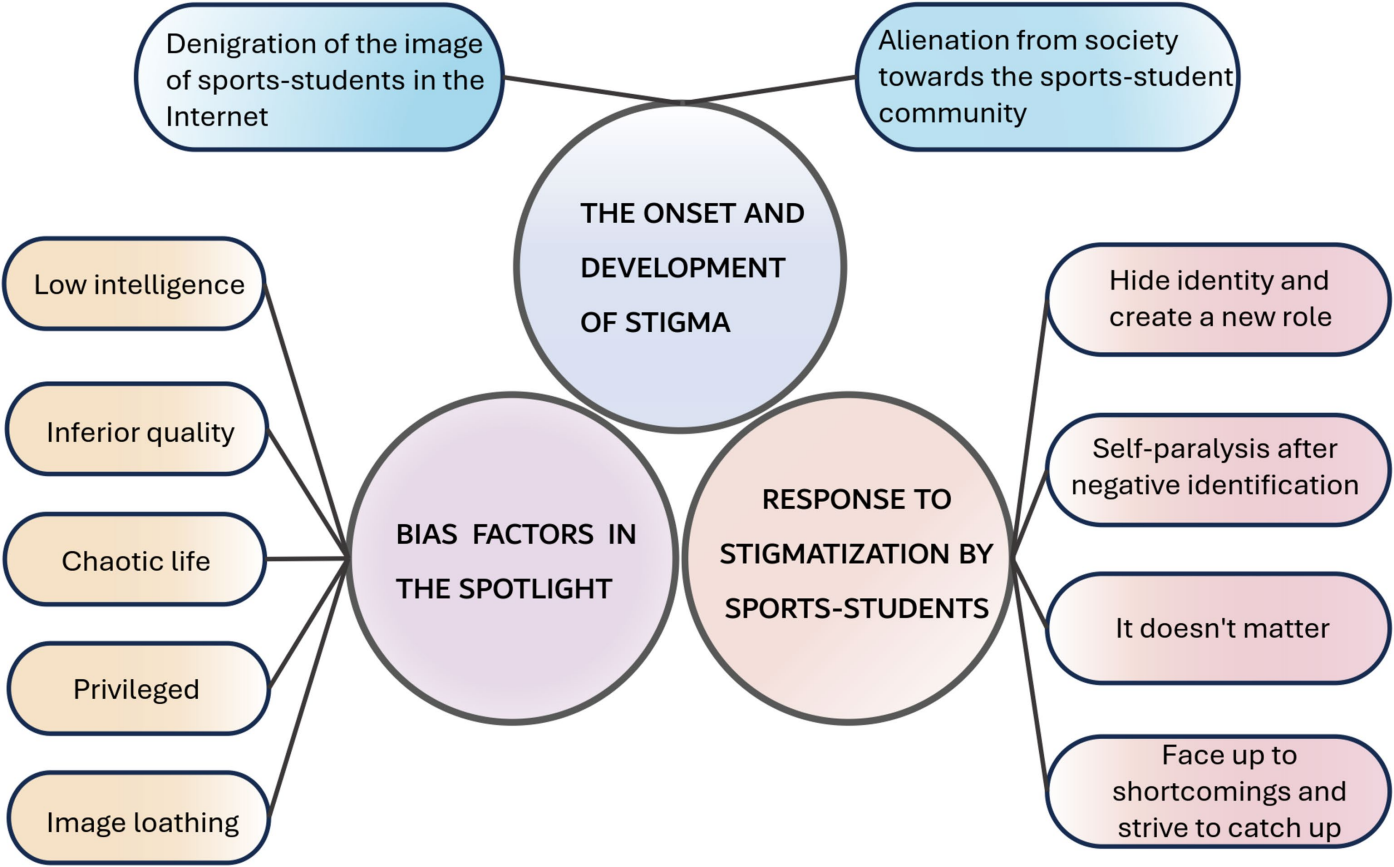
Final themes and sub-themes.

This research aims to understand and recognize the phenomenon of stigma associated with Chinese sports-students. Through investigation and analysis, a diagram was created to show the three themes and the sub-themes proposed by the research (Figure 1). The first theme describes the onset and development of stigma. The second one is about the bias factors that are spotlighted in stigmatization. The last is the response of sports-students to stigma.

### 4.1 The onset and development of stigma

Prejudice against sports has always existed in Chinese education, and most sports students with sports-related identities have experienced or are experiencing stigmas against them from other groups. These stigmas often occur online, on campus, and in their daily lives. Along with the stigma may come discrimination and alienation from different groups.

#### 4.1.1 Denigration of the image of sports students in the internet

Because of the anonymity of the Internet, the lack of regulation, and the difficulty of holding reality accountable, it has also become a hard-hit area for defaming sports students’ image. Most of the participants indicated that they had encountered sarcasm against sports students on the Internet:

*A lot of the videos and comments online carry topics like # Thanks Coach # ChenDian* (Accumulation) *# Stay Away from Injuries…but their content has nothing to do with sports at all; it’s all about ridiculing us by parodying some of the sports students’ copy…they see these words as a reflection of the sports students’ love of braggadocio* (HS1).

*In many of the videos with danger warnings, it is often commented, “Leave it to the sports students, they are not afraid to die”* (UG3).

It was also observed on the Internet that the keyword “sports students” was often associated with some sarcastic content. However, most of the publishers of these contents have yet to experience with sports students, and even fewer have been hurt by sports students. They do not try to understand the reality but find a bit of causal connection from other online works and put it in their content, then let more people see it, creating a vicious circle of negative stereotypes of sports students.

#### 4.1.2 Alienation from real world toward the sports-student community

As other studies have shown, Chinese sports students suffer from the same academic stigma on campus. This can lead to them being treated differently by their peers and teachers. Teacher 2, for example, spoke of how “*many teachers feel that even if they seriously ask them to study, the results are not good, and simply hope that the sports-students do not affect others*.” This neglect often affects the atmosphere of the entire class as well. Classmate 1 stated, “*It’s fine to be friends with them, but I guess it’s hard to create a crossroads in academics*.” These prejudices extend from high school to college, as sports students themselves know all too well:

*When working together on group work, if they find out it’s a group of sports-students, they opt out for fear of getting a low grade. And sometimes, if we are in a group with them, our opinions are not valued, which is really frustrating* (UG1).

This phenomenon of being academically disliked in college was concentrated among high-level athletes attending normal majors. Participants attending sports majors did not have a strong sense of this type of experience because their peers surrounded them. However, they had other troubles as well:

*I often feel ignored at some club events, and they seem not to want to talk to me because of who I am… when socializing, I may laugh at myself by using online mocking words about sports-students to find a topic of conversation with them* (UG4).

And people seldom get to know sports students better. In informal conversations, many average students said they had no contact with sports students, and their impressions of sports students came mainly from descriptions on the Internet or in other people’s words. Classmate 3 thought that “*they are often out training and competing, and do not have the chance to get in touch with them*.” On the other hand, parents of sports students seem to care little about their situation. Parent 1 argued that “*her (child) number one priority right now is to become a Division I athlete as soon as possible to attend a good college*.” Parent 2 suggested that, “*I’ve always thought that as long as he (child) stays out of trouble, gets into college by playing sports, and gets a steady job, that’s all that matters*. “But when it came to hiring, the researcher’s observations revealed that there were very few jobs that corresponded to sports majors, and recruiters for those jobs that did not restrict majors did not look favorably on the prospects of sports students. In response to all this negativity, from academic ability to workability, PG2 felt that “*the alienation from the real world is much more chilling than the true and false sarcasm online*.” The data from the informal interviews showed that these employers mentioned that the reasons for their mistrust centered on two main aspects: first, the low level of contact with the reality of sports students and the poor social evaluation of sports students, and second, the stereotypical belief that sports students do not do well academically and that their careers seem to be suited only to being PE teachers or fitness trainers.

### 4.2 Stigma factors in the spotlight

Participants felt that sports students are stigmatized in various ways, from inner qualities to outward appearance. Although many of these negative factors existed only in a few individuals, in a sociocultural context where there were various misconceptions about sports students, these prejudices were magnified until they covered the whole group.

#### 4.2.1 Low intelligence

“Dumb Jock” is a common negative stereotype of sports-students, and they carry the stigma of having low intelligence. HS3 mentioned, “*Whenever I have a conflict with someone, the first thing they do is call me limber and simple-minded*.” Compared with the general Gaokao, the academic scores required for university admission through sports are lower. The original intention of the relevant organizations in formulating this rule was to nurture more sports talents and balance the time and energy spent on sports training by lowering the score requirement. However, the reality is that most people would think that only students with low intelligence would engage in sports. Informal interviews with many regular students suggest that sports students turn to sports because they fail academically. This statement, however, is not true:

*My dad was a gym teacher, and he thought I had some athletic talent…they (parents) felt that I could go to a better college* (HS4).

*So many of us started practicing sports at a young age (this participant started at age 4) and did not have access to a systematic academic education like they (average students) did…I had teammates who were also very smart, ah, and went to college, worked hard, and could get scholarships through their academic performance, not athletic performance* (UG2).

Despite the many examples that can be disproved, it seems that stupidity has become the number one label for sports students underscores only. Participants loathed this bias, and UG4 felt that “*it’s the same as recognizing that we are always going to be dumber than everyone else, so why do we have to be inferior?*” As with previous research, the academic failure of sports students is of particular concern. Given the discrepancy between the rhetoric promoting academic supremacy and sports students’ educational experiences, this focus on adverse outcomes is understandable. However, many factors contributing to academic failure and attrition are not inevitable or intrinsic to sports students but are products of socialization (Haslerig, 2017).

#### 4.2.2 Inferior quality

Participants felt that people always tie sports-students to some inferior qualities. This subtheme captures some characteristics they mentioned, such as violence, bullying, impulsiveness, laziness, and lack of responsibility. HS3 said, “*We look fitter in sports, so they always think we fight a lot and bully them.*” HS1 also felt that “*many people compare us to ruffians.*” People around the sports students also carry some prejudice. In the informal interview, a student mentioned, “*Some sports students do often fail to hand in their homework, and I do not dare to rush him for fear that he will bully me behind my back.*” Participants perceived most of these described traits as group biases triggered by individual behavior:

*My parents and coaches taught me from a young age not to use violence to solve problems, much less bully others…many people just map the violence of professional games and conflict onto us…at the end of the day, we are still students, and pretty friendly teammates surround me* (UG1).

*Sometimes, training does get too tiring for me to resist sleeping (in class)… We participate in class activities much more actively than they (average students) do* (HS4).

The researcher’s observation indicates that these so-called traits genuinely exist in a few individuals, while most sports students behave no differently than regular students. Most of them maintain a cheerful, optimistic mindset and positive attitude due to sports. However, in informal interviews, some people still say that sports students are the representatives of “bad students.” Some negative stereotypes have been ingrained in some people’s minds for a long time, and even when they come across some of the good qualities of sports students, it seems that these people still feel that the good ones are the exception and the bad ones are the norm.

#### 4.2.3 Chaotic life

Participants indicated that keywords such as smoking, alcoholism, and promiscuity often appeared in people’s negative evaluations of sports students’ lives. UG3 mentioned, “*People always think that I have a lot of girlfriends, but in fact, I’ve only been in love twice.*” The stigma of promiscuity is indeed prejudicial. And in the researcher’s observation, the daily life of sports students is more relaxed than described. They would drink alcohol a little more frequently than the average students, but not heavily. UG4 stated, “*Sometimes, after finishing a training session or a competition, it is always a good idea to get together for a drink to release stress or to celebrate.*” But there is always an indistinguishable correlation between sports and alcohol consumption, and it cannot be arbitrarily labeled as bad (Palmer, 2014). Some participants were averse to smoking themselves; for example, HS1 said, “*The coach would have to yell at me if I wanted to smoke, and it would affect my athletic performance.*” However, the researcher’s reflective journal mentions that the smokers in the observation mainly existed in small groups, i.e., if one person smoked, there was a high probability that his friends around him also smoked and vice versa. And this does not only apply to sports students, but to other student groups as well.

#### 4.2.4 Privileged

In the view of others, sports students seem to enjoy many privileges. In informal interviews, some high school students expressed to the researcher how much they envied sports-students not having to attend morning and evening study sessions, whereas normal students were penalized if absent. In response to this privilege, HS2 referred to, “*We have early morning training, and in the afternoon, we all go back to training after classes, plus we have to relax, shower, and eat, so we do not have time for self-study at all.*” The researcher’s online observations revealed that there are often anonymous users in the forums who slam sports students for hogging the use of the school’s sports facilities and also charge that it is an unfair privilege given to them by the school. UG5 mentioned, “*We are only allowed to use those facilities freely when we are taking our specialized classes, and we have to make reservations for the rest of the time as well…the school should be made to provide more resources for sports.*”

Furthermore, the so-called “academic privilege” is of most significant concern. Both participants who were not majoring in sports stated that they had heard rumors that they did not have to work hard to get high grades:

*I had average grades, and my teachers never gave me any special attention…my teammate ranked very high on a final exam once and got a scholarship. Still, there was always gossip in the class that she got high grades by pleasing her teachers and her athletic performance…they just hit the heart that we would not study hard* (UG2).

*They would always ask if our GPAs included sports scores and if we were taking up a guaranteed spot in graduate school…it made me feel awkward studying the same together as if we were aliens* (UG1).

In response to this stigma, Teacher 3 addressed, “*I have not gone out of my way to take special care of the grades of the sports students, and our teachers have not been notified that we are to be lenient with them in their studies…The school is supposed to have special rules for calculating sports grades, and I’m not sure.*” Be that as it may, the stigma still will not go away from the truth as long as the conflict of interest between the parties involved remains.

#### 4.2.5 Image loathing

**Figure 2:**
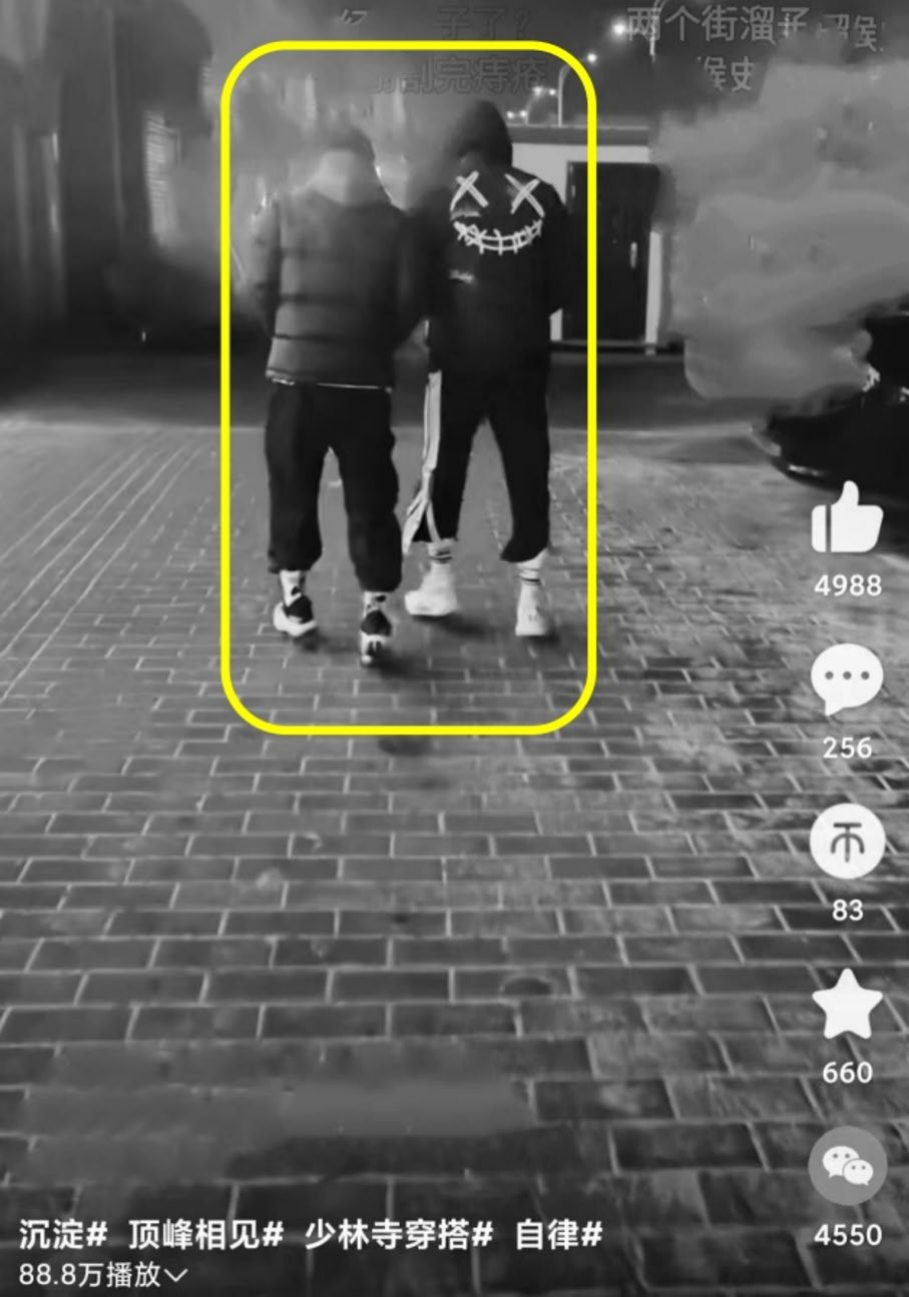
Screenshot of the video provided by the participant (relevant privacy information has been blurred).

In addition to those intangibles being evaluated negatively, the stigma has even grown to the specific outward image of sports students:

*There are often satirical videos on the internet with the theme “Teach you how to recognize a sports student” that describe male sports students who like to wear socks over their pants, walk on their tiptoes, and sway their bodies from side to side (*Figure 2*)… female sports students who wear tight yoga pants… Anyway, I do not intentionally pick my outfit now…I’ll change back into regular clothes right after a gym session* (PG1).

Most sports students’ daily routines did not present distinctive images and behavioral characteristics in the observation. HS1 mentioned, “*It’s so tiring to train all day long; where do you find the time to get that whole thing…There are people around me who dress like that, but it seems to be pretty poorly regarded online*.” The researcher’s reflective journal describes this image loathing as stemming from the mockery of the “cyber sports student,” a term used to refer to authors who advertise themselves as sports students in their online work, which consists of a display of outfits and behaviors that are not associated with sports (sort of a show-off). These online depictions of dress or behavior patterns, mixed with stereotypes of sports students, have evolved into one of the leading labels for the outward image of realistic sports students. Still, the label expresses a mockery of this identity, according to UG3: “*We cannot argue with it… sometimes, we accidentally clash with the outfits depicted online. We also laugh at ourselves for wearing an ‘Accumulation suit’ (derision of such outfits) today.*” The sports students show more helplessness to such taunts; they cannot do more, and if they go for a rebuttal, it may lead to another round of special attention.

### 4.3 Response to stigmatization by sports students

Stigmatized participants’ responses to stigma vary, but both positive and negative coping mechanisms reflect the fact that the stigma in question truly works on the victim. This theme summarizes the responses of sports-students to stigma.

#### 4.3.1 Hide identity and create a new role

The identity of sports students is valued when they are in the field of sports; for example, HS4 referred to, “*Every year when the school sports meet is the time when I am at my highest because I can get a lot of honors for my class*.” However, their identity may be belittled or even stigmatized when it is placed in other fields, such as academics, and some of them would be ashamed to admit this identity. UG5 said, “*I rarely mention my identity to others, and if I tell some relatives that I majored in sports, they may think I have no future.*” That is why some people are willing to hide their identity so that they do not have to care about the negative perceptions associated with sports students:

*If people do not ask me, I will not tell them (my identity), I do not like to be called a sports student… I take the same classes and the same papers as them (average students), Whenever I mention “sports student,” (average students) feel I am taking advantage of them* (UG1).

*On some informal or unfamiliar occasions, I will say that I study other majors (non-sports). I said I was a graduate student, and he would think it was very impressive; when I said I was a sports major, I immediately felt that he had a kind of disdain for the expression… Anyway, as soon as a driver asks, I’ll say I’m a graduate student in law… This feeling of being respected is great* (PG2).

Social Identity Theory suggests that individuals will increase their self-esteem by maintaining a positive social identity. Individuals will use strategies to improve self-esteem when their social identity is threatened. If the inferiority of their group cannot be denied, then people will try to leave their current group and join a more favorable group (Stets and Burke, 2000). A similar behavior was seen in the study by a participant who went for a good experience by creating a new role for himself and attributing his identity to a solid out-group to avoid some of the devaluation and conflict.

#### 4.3.2 Self-paralysis after negative identification

In addition to the impact of stigmas from the outside world, the results of the study show that some sports students internalize those stigmas and thus paralyze themselves by demanding less of themselves or avoiding challenging tasks:

*Sometimes when assigning after-school tasks, some of the sports students would say to me, “Teacher, give me some simple tasks, I cannot study anyway, so I do not want you to worry…” It’s kind of frustrating, and it’s like they are limiting themselves to that* (Teacher 2).

*When I instructed him (the child) to learn more about academics, he said he could not do the academics anyway, so he might as well go practice more for the sports test* (Parent 2).

Some participants also stated that they sometimes use those labels as an excuse for themselves. HS3 said, “*Sometimes it is normal to think that you are not good at academics and that it is enough if you can be good at sports.*” In addition to academics, other areas may be less demanding because of who they are. In informal interviews with a handful of high school sports students who smoke, some of them mentioned, “*It’s normal for us (sports students) to smoke, and no one else will have a problem with it*.” This behavior of internalizing the stigma and paralyzing themselves may harm their studies and lives. Particularly for sports students who are on campus, these self-stigmatizing behaviors may further exacerbate negative external perceptions of this group.

#### 4.3.3 Face up to shortcomings and strive to catch up

Those stigmas create a terrible experience, but some participants show a positive attitude to cope with them. They may reject stigmas by trying not to cater to those wrong impressions through methods such as studying hard and improving their qualities. Although they did not share the same views on stigmas, they all had a positive attitude toward learning and living. Several participants indicated that some stigmas are heard so much that they subconsciously compare these perceptions on themselves to make themselves not annoying to others:

*I often take note of other people’s strengths in the club and want to be as good as they are…When I hear negative opinions about us (sports students), I also look at my weaknesses and hope to do what I can to correct them* (UG4).

Some participants believed that all people have equal status and should not feel free to judge others by some so-called standards. For instance, UG1 said, “*I do not think I’m any different from them; they also have shortcomings, and I believe I can do better.*” Some sports students also work hard in the eyes of others; Classmate 4 mentioned, “*Some sports students in my class do study quite hard and will often come to discuss questions with me.*” Most of these sports students in this study who expressed positive attitudes toward stigma were at the undergraduate stage, which may be related to the fact that they become more mentally mature as they grow older and the changes in the humanistic environment in which they are living, e.g., high school in China may be primarily aimed at teaching students how to learn. In contrast, at university, it involves more inspirational ideas about life and the value of life. Such changes need to be further explored.

#### 4.3.4 It does not matter

Another thing that some sports students expressed to the researcher was that the stigmas do not affect them. UG3 stated, “*What else can I do? I cannot change anything…do I need to reinvent myself? I’ve always been like this, I’m not doing anything illegal, I’m not a saint*.” And even if they cared about these negative perceptions, there seemed to be timeliness and spatiality. For example, UG2 mentioned, “*Sometimes when I hear those negative things, I always get righteously angry and want to do something, but I always forget about it after a while and feel that it does not matter*.” HS2, on the other hand, thought, “*I’m too busy now in my senior year of high school to take care of those (stigmas), so I should just do what I want…*.”

The reflective journal talks about this, and it is impossible to accurately determine the pros and cons of this attitude in a stigmatized situation. However, it is inevitable that they certainly chose to be brave amid the ins and outs. These participants expressed their indifference, but through observation during the exchange, it was not that they did not want to make changes to counteract the stigma; it was just that at the student stage, they had too many other things to do, and the most they could do was to minimize the impact of these bad perceptions on themselves, and selectively ignore some of the stigmas from the outside world.

## 5 Discussion

This study aims to understand and recognize the phenomenon of stigma suffered by sports students in China. The findings suggest that sports students are a group that is being stigmatized, which is the same as other studies of student-athletes. These stigmas fester and spread on the Internet, creating alienation from the sports student group. In addition to the typical stigmas, the study identified new focal points, such as a distaste for the image of some sports students. The alienated group can truly perceive these alienating views from other groups and respond in various ways, with an interesting point being that some participants said they would give themselves another layer of identity to get a good experience. The so-called stigma associated with this group is nothing more than a way for other groups to maintain the dominance of the in-group by covering the entire group of sports students with the human inferiority of some individuals (Al Ramiah et al., 2011).

The study found that sports students were stigmatized about personality and academics, similar to previous studies (Simons et al., 2007; Stone et al., 2012). How stigmas are perceived and treated by others can significantly impact stigmatized individuals, and these stigmas may form the basis of the perceiver’s negative expectations of behavior (Goffman, 1986). These expectations can reinforce the perceiver’s sensitivity to stigma-related behaviors. Behaviors that may typically occur in various groups stand out when the perceiver confirms the expectations of the stigmatized group. For example, it has been found that while negative academic behaviors may not occur more frequently in student-athletes than in non-athletes, these behaviors become more salient in the minds of faculty and staff due to negative expectations of student-athletes (Simons et al., 2007). So, others discover the terrible aspects of individual sports students. In that case, these impressions become evidence that justifies the negative expectations of this group, which in turn overrides the conclusions of the entire group. Moreover, the derogatory and insulting labeling of these individuals or groups will serve as a starting point for social discrimination (Goffman, 1986), resulting in the alienation of sports students.

### 5.1 Background and motivation for stigmatization

For stigma inquiry, we must emphasize the contextual nature of stigmatization and the specificity of the perspective of understanding, as well as focusing excessively on the individual while ignoring the socio-cultural contexts and processes that affect the individual (Link and Phelan, 2001). Chinese society has regarded Confucianism as the core of morality and social harmony for thousands of years, while Confucianism has dominated the content of traditional Chinese education, with most parents using it as a guide to their parenting practices. However, Confucianism has placed far more emphasis on education than physical activity (Lau et al., 2007). One can get a glimpse of this from the rules on children’s behavior in the Confucian classics, where childish traits and naughtiness are discouraged or even prohibited in the mature Confucian norms. In other words, to a large extent, traditional Chinese education aims to mold the “ordinary child” by the Confucian image of the “ideal child,” hoping to present an image of quietness and refinement. At the same time, sports of an active and playful nature are not promoted (Bai, 2005).

Furthermore, Chinese cultural traditions consciously differentiate between “*wen*” and “*wu*,” where “*wen*” refers to the use of the mind, while “*wu*” focuses on the body and requires physical strength. Because of the civil service examination system, which was put into effect during the Sui dynasty, the idea that “everything is low-class work except academic study” (*wanban jie xiapin wei you dushu gao*) permeated Chinese society (Yu and Bairner, 2011). The concept of emphasizing civilization over the military (*zhongwen qingwu*) has also impacted the culture of various dynasties (Lorge, 2000). Nevertheless, it is undeniable that this idea not only undervalues physical activities such as sports but also serves to stigmatize parts of the population in some societies (Yu and Bairner, 2011). Studies have also shown that sports students are often categorized as “dumb” and “uneducated.” In this traditional culture, they are also more likely to be prejudiced and discriminated against to varying degrees. Moreover, those who are highly intelligent, educated, and talented are often given a positive social image and are more likely to have a higher social status and prestige in the future. In contrast, the image of the opposite group is always relatively negative, and future development is not expected (Yopyk and Prentice, 2005).

Some social systems that have existed for a long time may contribute to stigma out of inertia due to the formation of taken-for-granted cultural norms. For example, the Gaokao system, which has been in force in China for many years, is the traditional Chinese way of talent cultivation, leading the Chinese to emphasize academic success more (Guo, 2023; Lecheng, 2022). Meanwhile, because Chinese universities generally admit students only based on Gaokao scores, this system maintains the dominant social value of test scores being the most important (Muthanna and Sang, 2016), which in turn profoundly influences the acquisition of values in the socialization process of many people, and may lead to biased understandings of sport and sport-related identities. In current Chinese schooling, most students are admitted to universities based on academic performance. Thus, students with good academic performance and their parents, teachers, and other groups hold the power of discourse (Yu et al., 2018). Social identity theory suggests that to maintain a sense of superiority associated with group membership, people tend to compare their in-group more favorably with other out-groups (Tajfel, 1969). Therefore, as representatives of the dominant values in the social culture, the “Gaokao group” cannot allow others to question the concept of “scores first,” which is their favorable identification with their group. Such groups, in a sense of superiority, tend to maintain their status with prejudiced beliefs. As demonstrated in previous research, although the average student knows that there are student-athletes who excel athletically and academically, they also consistently refer to student-athletes with negative attributes, such as minimal academic effort (Yukhymenko-Lescroart and Sharma, 2022). This highlights a form of social comparison that elevates the values of the in-group for academic pursuits over the perceived values of the sports students.

Another possible stigma-motivated explanation is realistic group conflict theory, which argues that bias occurs once groups compete for scarce resources (Zárate et al., 2004). Hostile prejudice against sports students is most substantial among those whose academic performance is closest to that of the sports students in the Gaokao, and prejudice becomes a means of retaliation when there is a conflict of interest (Li, 2014). Besides, there is always the perception that student-athletes are admitted to colleges with lower academic standards than the general student population and are seen as being “pushed ahead” and having too much privilege (Umbach et al., 2006). The narratives in this study also refer to biases related to privilege, and these so-called “privileges” are primarily the result of the collision of interests of the parties involved. For example, “academic privilege” was perceived in conversations primarily by sports students enrolled in general majors because they need to be compared to general students. In contrast, undergraduates in sports-related majors seldom reported such situations. Even though many of the same resources, such as tutoring, are available to athletes and non-athletes alike, traditional students perceive this as unfair and believe that sports students receive special treatment rather than the grades they deserve (Ross and Shinew, 2008).

### 5.2 The internet’s exacerbation of stigma and the plague of intersecting identities

Furthermore, the easy accessibility and virtual nature of the Internet are highly susceptible to frivolous public opinion attitudes, which has led to the Internet gradually becoming a breeding ground for stigmas. Other studies have also shown that one of the main reasons behind these stereotypes is the pervasive influence of negative images of student-athletes on social media (Yukhymenko-Lescroart and Sharma, 2022). Overreporting some bad news can exacerbate negative perceptions of this group (Haslerig, 2017). A low critical mass and rapid diffusion characterize these online stigmatization phenomena. For instance, the climax of the alienation of the image of sports students on the Chinese Internet is fermented in some online videos about their outfits or behaviors. However, it is essential to emphasize that there is nothing morally or legally wrong with these outfits *per se*. Wearing socks, for example, is a fashionable way of dressing worn by many sports stars and fashionistas. What people loathe is the hypocrisy in a quick-fix identitarianism: with a few fixed clothes, a fixed walking posture, a soundtrack, and no accomplishments or proofs required, the average person can rock up and get an identity card. Secondly, there is a perception that this identity is emphasized for bragging rights purposes, which overlaps with other prejudices about sports students. This ultimately creates a two-way corroboration of image and identity for this group, whereby a lousy image represents a specific identity, and a fixed identity is perceived to come with a negative image. The anonymity of the online virtual society and the weakening of social norms and ethical constraints have led some online users to mock this figurative identity. This includes the element of herding because once prejudices are formed and accepted by society, more people will follow the most unobstructed path, conforming to the trend to win the favor of the group or to differentiate themselves from the group they are targeting (Yukhymenko-Lescroart and Sharma, 2022).

Identity reflects the order and position of people in the social structure, a normative guideline for measuring each other and orienting interpersonal relationships, and a group category defined by the distinguishing characteristics that members of a society take pride in (Caza et al., 2018). Those stigmatized sports students in this study appeared to have two distinct identities, where the student identity was valued, and the sports identity always seemed to be prejudiced. The stigma is context-dependent because a person’s social identity is devalued in one context but not another. For instance, an athlete’s athletic identity is stigmatized in the academic realm but highly valued in the athletic realm (Simons et al., 2007). This is why some sports students hide their sports-related identities in certain situations to prevent being categorized as outliers. In addition to external compartmentalization, the interaction of these two identities can be troubling for themselves. A study indicated that college athletes are more susceptible to psychological distress when the student role is not differentiated from the athlete role because negative experiences in one role are carried over into the other (Settles et al., 2002). For example, research suggested that athletic identity or negative athletic performance may interfere with a student-athlete’s academic ability (Stone et al., 2012; Fuller et al., 2017). This could also explain some participants’ expressions that sports students are no worse than regular students and can even surpass them. Because without the time constraints and physical demands of athletic training, they would perform just as well as the average person (Singer, 2008).

### 5.3 Impact of stigmatization

Social identity threat due to group activation of negative group stereotypes or stigmatization may impair people’s social quality of life (Martiny and Nikitin, 2019). Some of the sports students in this study would internalize those stigmas and thus paralyze themselves; they would demand less of themselves or avoid challenging tasks. Corrigan argued that stigma is an integrated whole consisting of public stigma and self-stigma. Public stigma is the derogatory stereotyping of particular stigmatized groups by generalized social groups, while self-stigma is the self-depreciation and self-discrimination that follow public stigma (Corrigan, 2004). Thus, besides the impact of stigmas from the outside world, research findings demonstrate that some sports students internalize those stigmas and paralyze themselves by holding themselves to lesser standards or avoiding challenging tasks. Other research also suggested that student-athletes may believe that they lack the intellectual capacity to succeed academically. This belief can become a prophecy that hinders self-fulfillment, causing them to essentially internalize the “dumb jock” stereotype and attempt to avoid or resist academic situations (Steele, 1992).

The study also found that while sports students lost their identification with academics, they also increased their identification with sports. This phenomenon may lead them to devote more time and energy to sports, strengthen their ties with peer groups, and look down on regular students as “nerds.” Identity is then reorganized, and their self-worth becomes entirely dependent on the field of sport (Simons et al., 2007). This can further increase the social distance from the general student population, and the lack of contact between the two groups is likely responsible for some of the stereotyping (Yukhymenko-Lescroart and Sharma, 2022). The study also revealed that self-stigmatization seemed to improve after encountering virtual spaces; for example, some participants reported that they also flirted with their sports identity online, which was not an identification with public stigma but more of a recreational form of self-stigmatization. The cyberspace provided a venue for them to escape their negative emotions, which gradually diluted the substantive content of some stigmatized people’s sense of self-depreciation. Thus, the relationship between self-stigmatization and public stigmatization associated with sports students is no longer the traditional concomitant relationship in the virtual space, which is yet to be fully understood.

Stigmatized individuals are often subject to stereotype threat, which can be generated by simply reminding individuals of their stigmatized social identity (Hsu and Li, 2021). This can negatively affect the target, who will be threatened regarding perceptions, beliefs, attitudes, and academic ability (Stone et al., 2012; Riciputi and Erdal, 2017). However, it has also been suggested that stereotype threat may lead to the opposite response, i.e., the threatened target should be highly motivated to perform well on the task to overcome the negative portrayal (Steele and Aronson, 1995). Nevertheless, if the threat depletes the cognitive and other resources needed to accomplish the task, they may be unable to overcome the burden by increasing their effort (Schmader and Johns, 2003). Consistent with the previous research demonstrated (Harrison et al., 2009), this hypothesis is supported by the descriptions of some of the threatened sports students in this study who felt that they had tried hard. However, the results were sometimes different from what they had hoped for.

Additionally, research has shown that some participants preferred to display a student identity or even create a respected identity while avoiding a sports identity, which may also be a strategy to cope with threats. In such a situation for individuals with multiple group identities, an effective strategy for eliminating stereotype threat is switching group identities, i.e., switching from a negative to a positive group identity, which aims to eliminate the cognitive dissonance that triggers the threat (Schmader et al., 2008). Some studies are also actively exploring strategies to mitigate this threat, such as focusing such groups on alternative identities to reduce barriers to academic engagement (English and Kruger, 2020); in addition, developing mental toughness may be a way to increase their wellbeing and reduce the stigma of seeking help (Bird et al., 2021).

### 5.4 Implications and limitations

The study results are expected to bring more attention to the real information about this group instead of going with the flow of stigmatization to drown out the truth. The group of Chinese sports students has its peculiarities, but they are still displaying the role of students primarily; sports should not have been stigmatized in the first place, and sports-related identities should not be a reason to be biased against them. Like all students, sports students are multifaceted and committed to exploring opportunities to define their academic, professional, and personal paths (Haslerig, 2017). Other scholars have already called for a reform of the term student-athlete, arguing that if the term exists, there should also be terms such as student musician, student artist, or student engineer (Staurowsky and Sack, 2005). Similarly, in China, where education is diverse, “sports students” should not be singled out for judgment.

Simultaneously, educational institutions should also promote changes to help de-stigmatize sports students. For example, rules and regulations related to sports facilities and students’ academic performance should be open and transparent to reduce suspicion of privilege; secondly, intergroup contact reduces prejudice (Pettigrew et al., 2011), so interactions between different groups of students should be facilitated to increase understanding of sports students by lowering group boundaries and increasing inclusiveness; and to help integrate sports students into the community through the creation of diversified employment opportunities; Corresponding psychological support should also be given to sports students to avoid self-paralysis and negative coping brought about by stigma. In short, this is a systematic project that requires the attention of administrators.

The findings should also be considered in light of their limitations. Specifically, due to the complexity of China’s educational environment, we do not have a comprehensive understanding of what happens to sports students in some areas; for example, the researcher learned that some high schools divide their sports students into separate classes for teaching, and the pros and cons of such a model are yet to be fully understood. In addition, what was obtained through the one-time interviews in the research design was a snapshot of participants’ perceptions of stigma at the time. Future research may also adopt a longitudinal approach to the study to explore how sports students’ understanding of stigma changes over time and in their environment and how they find themselves amid such changes.

## 6 Conclusion

This study aims to understand and recognize Chinese sports students’ experiences of stigma and their perceptions of this. The study suggests that sports students generally suffer from stigma related to the traditional Chinese culture of valuing civilization over the military and the college entrance examination system in which academic performance holds the power of discourse. At the same time, people’s stereotypical image of sports identity also contributes. These implicit factors and the fermentation of some online events have added negative filters to the identity of sports students in society. Among the common stigmas come from the negative portrayal of sports students in terms of intelligence, quality of life, and authority. The findings also provide new insights, such as image loathing festering mainly on the internet, where people mock some so-called sports students’ outfits or behavior. This may indicate that the internet is a vital enabler in stigmatizing modern sports students. The study also found that the effects of stigma have permeated groups of sports students in different school years and at various sports levels. Of note, stereotype threat and self-paralysis following self-stigma may induce harmful consequences. These findings will provide evidence-based support for subsequent research on the de-stigmatization of sports students.

## Data Availability

The raw data supporting the conclusions of this article will be made available by the authors without undue reservation.
